# Risk factors for severe COVID-19 in middle-aged patients without comorbidities: a multicentre retrospective study

**DOI:** 10.1186/s12967-020-02655-8

**Published:** 2020-12-07

**Authors:** Peng Wang, Jing Sha, Mei Meng, Cuiyan Wang, Qingchun Yao, Zhongfa Zhang, Wenqing Sun, Xingguang Wang, Guoqiang Qie, Xue Bai, Keke Liu, Yufeng Chu

**Affiliations:** 1 Department of Critical Care Medicine, Shandong Provincial Hospital Affiliated to Shandong First Medical University, No. 324 Jingwu Road, Jinan, P. R. China; 2 Department of Critical Care Medicine, Ruijin Hospital, Ruijin Hospital North, Shanghai Jiao Tong University School of Medicine, Shanghai, P. R. China; 3 Shandong Medical Imaging Research Institute Affiliated To Shandong University, Jinan, P.R. China; 4 Jinan Infectious Diseases Hospital, Shandong University, Jinan, P. R. China; 5 Department of Intensive Care Unit, Shandong Provincial Chest Hospital, Jinan, P. R. China; 6 Department of Pulmonary and Critical Care Medicine, Shandong Provincial Hospital Affiliated to Shandong First Medical University, Jinan, P. R. China; 7 Shandong Academy of Clinical Medicine, Shandong Provincial Hospital Affiliated to Shandong First Medical University, Jinan, P. R. China

**Keywords:** Coronavirus, COVID-19, Middle-aged, Risk factors, SARS-CoV-2

## Abstract

**Background:**

Information regarding characteristics and risk factors of COVID-19 amongst middle-aged (40–59 years) patients without comorbidities is scarce.

**Methods:**

We therefore conducted this multicentre retrospective study and collected data of middle-aged COVID-19 patients without comorbidities at admission from three designated hospitals in China.

**Results:**

Among 119 middle-aged patients without comorbidities, 18 (15.1%) developed into severe illness and 5 (3.9%) died in hospital. ARDS (26, 21.8%) and elevated D-dimer (36, 31.3%) were the most common complications, while other organ complications were relatively rare. Multivariable regression showed increasing odds of severe illness associated with neutrophil to lymphocyte ratio (NLR, OR, 11.238; 95% CI 1.110–1.382; p < 0.001) and D-dimer greater than 1 µg/ml (OR, 16.079; 95% CI 3.162–81.775; p = 0.001) on admission. The AUCs for the NLR, D-dimer greater than 1 µg/ml and combined NLR and D-dimer index were 0.862 (95% CI, 0.751–0.973), 0.800 (95% CI 0.684–0.915) and 0.916 (95% CI, 0.855–0.977), respectively. SOFA yielded an AUC of 0.750 (95% CI 0.602–0.987). There was significant difference in the AUC between SOFA and combined index **(**z = 2.574, p = 0.010).

**Conclusions:**

More attention should be paid to the monitoring and early treatment of respiratory and coagulation abnormalities in middle-aged COVID-19 patients without comorbidities. In addition, the combined NLR and D-dimer higher than 1 μg/ml index might be a potential and reliable predictor for the incidence of severe illness in this specific patient with COVID-19, which could guide clinicians on early classification and management of patients, thereby relieving the shortage of medical resource. However, it is warranted to validate the reliability of the predictor in larger sample COVID-19 patients.

## Background

The novel coronavirus disease 2019 (COVID-19) pandemic has posed a great health threat globally. As of June 16, 2020, according to the latest situation report from WHO, the SARS-CoV-2 has infected 7,823,289 people around the world and caused 431,541 deaths [1]. The population is generally susceptible to the SARS-CoV-2. Unfortunately, much of the pathogenesis and optimal therapy of COVID-19 remains unclear.

Rapidly accumulating evidences have shown the risk factors for severe illness and death in COVID-19. Based on current studies, older age has been identified to be associated with an increased risk of death in COVID-19, as well as comorbidities [2–6]. The presence of these comorbidities might have increased the risk of mortality independent of SARS-CoV-2 infection. A meta-analysis of seven studies including 1576 patients with COVID-19 indicated that those patients with severe illnesses were more likely to have hypertension (odds ratio 2.36 [95% CI 1.46 to 3.83]), respiratory disease (2.46 [1.76 to 3.44]), and cardiovascular disease (3.42 [1.88 to 6.22]) [7]. However, the deterioration or death of these patients might attribute to not only SARS-CoV-2 infection, but also the originally damaged organ function or the aggravation of underlying comorbidities induced by viral infection. Therefore, we presumed that the clinical manifestations of non-elderly patients without pre-existing diseases are closer to the real conditions of patients infected with SARS-CoV-2. As yet, risk factors and predictors for severe illness in this specific population have not been well described.

In the past few months, numerous studies have reported that different from young patients, a certain proportion of middle-aged patients developed into severe illness or even death after SARS-CoV-2 infection. The patients aged 40 to 59 years had a 1.4% case-fatality rate (CFR) in Italy [8] and 1.7% in China [9], respectively. And more importantly, as the backbone of society and family, more attention should be paid to middle-aged patients with COVID-19 for early identification of risk factors associated with poor outcomes. At the same time, the high-risk patients should be given strengthened monitoring and treatment so as to reduce the occurrence of critical illness and to decrease the mortality rate. It will not only help to understand the real harm of the SARS-COV-2 to human beings, but also to stabilize the society and family.

Herein, we described the details of clinical characteristics and outcomes of 119 middle-aged patients without underlying diseases from three hospitals designated for the treatment of COVID-19 in China, and explored risk factors and prognostic indicators for the severity of COVID-19.

## Methods

### Study population

The multicenter retrospective study was conducted at three hospitals designated for the treatment of COVID-19, including Jinan Infectious disease Hospital in Shandong, Shandong Provincial Chest Hospital in Shandong, and Huanggang Central Hospital in Hubei. The recruitment period was from January 31, 2020, to April 17, 2020. All patients enrolled in this study had received a diagnosis of COVID-19 according to the diagnostic criteria from the fifth edition of the Guidelines on the Diagnosis and Treatment of COVID-19 by the National Health Commission of China [10]. The presence of SARS-COV-2 in respiratory specimens was confirmed using real-time reverse-transcriptase polymerase chain reaction (RT-PCR) assay were performed in accordance with the protocol described previously [11]. The patients with comorbidities, pregnant women and patients younger than 18 years old were excluded. As of April 17, 2020, all included patients were discharged or died.

### Data collection

Demographic, comorbidities, clinical, laboratory, imaging examination, treatment, and outcome data were collected using a standardized case-report form. For patients with a readmission during the study period, data from the first admission are presented. All clinical outcomes were presented for patients who completed their hospital course at study end (discharged alive or dead). The Sequential Organ Failure Assessment (SOFA) score were calculated separately using the worst value of physiological variables within 24 h of presentation. All data were checked by two physicians (QY and PW), and then a third researcher (YC) determined any differences in interpretation between the two primary reviewers.

### Definitions

Severe COVID-19 was defined as ICU admission, respiratory failure requiring mechanical ventilation, use of vasopressor therapy, use of continuous renal replacement therapy (CRRT) or ECMO, or death. Comorbidity was defined as having at least one of the followings before diagnosis of COVID-19: hypertension, diabetes mellitus, coronary heart disease, stroke, hyperlipemia, bronchiectasis, asthma, chronic lung disease, chronic kidney disease, chronic liver disease, cancer, hematologic disease, autoimmune disease and HIV or other virus infection. Fever was defined as axillary temperature of at least 37.3 °C. Sepsis and septic shock were defined according to the 2016 Third International Consensus Definition for Sepsis and Septic Shock [12]. Acute liver injury was defined as the peak values of serum alanine aminotransferase (ALT) above threefold of the upper limit of normal (ULN). Acute kidney injury was diagnosed according to the KDIGO clinical practice guidelines [13] and acute respiratory distress syndrome (ARDS) was diagnosed according to the Berlin Definition [14]. Acute cardiac injury was diagnosed if serum levels of cardiac biomarkers (e.g. high-sensitive cardiac troponin I) were above the 99th percentile upper reference limit, or if new abnormalities were shown in electrocardiography and echocardiography [11].

### Statistical analysis

The Kolmogorov–Smirnov test or Shapiro–Wilk test was used to test the normality of the continuous variables. Continuous variables of normal distribution were expressed as mean ± SD and compared using the unpaired, 2-tailed student’s t test. Continuous variables of skewed distribution were showed as median (interquartile range) and compared with Mann–Whitney U test. Categorical data were summarized as number (percentage) and compared by χ^2^ or Fisher’s exact test. To explore the risk factors associated with the risk of progression to severe disease or death, logistic regression analysis was conducted to estimate OR and 95% confidence interval (95% CI). Considering the total number of severe cases (n = 18) in this study and to avoid overfitting in the model, two variables were chosen for multivariable analysis on the basis of previous findings and clinical constraints [2, 15–17]. We excluded variables from the univariable analysis if their between-group differences were not significant, if the number of events was too small to calculate odds ratios, and if they had colinearity with the SOFA score. Additionally, the receiver operating characteristic (ROC) curves were analyzed by ROC package to evaluate the performance of selected factor in predicting the development of severe COVID-19. The area under the curve (AUC) and 95%CI were derived from the ROC curve**,** and the optimal threshold for each selected factor and combined index was determined when the Youden index achieved the highest value. The differences between the AUC were detected by Delong’s test, which was a non-parametric approach and could generate an estimated covariance matrix by using the theory on generalized U-statistics [18]. A two-sided α of less than 0.05 was considered statistically significant. Statistical analyses were done using SPSS software, version 22.0 (SPSS Inc. Chicago, Illinois, United States) and R 3.6.2 (R Foundation for Statistical Computing).

## Results

### Demographic profiles and clinical characteristics of the patients with COVID-19 on admission

**Figure. 1:**
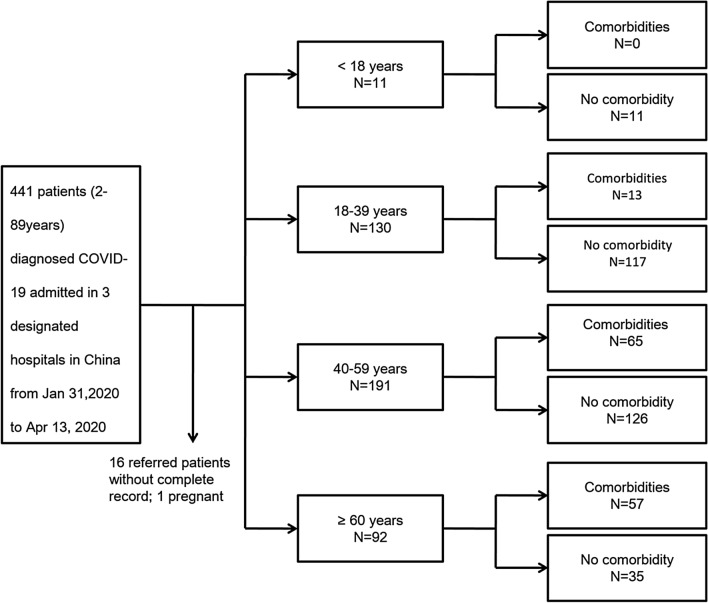
The incidence of comorbidities in different age groups for all 441 patients with COVID-19. Comorbidities were defined as having at least one of the followings before diagnosis of COVID-19: hypertension, diabetes, coronary heart disease, stroke, hyperlipemia, bronchiectasis, asthma, chronic lung disease, chronic kidney disease, chronic liver disease, cancer, hematologic disease, autoimmune disease and HIV infection

A total of 441 COVID-19 patients (range, 2–89 years) were hospitalized in the three designated hospital from Jan 31, 2020 to Apr 17, 2020. After excluding one pregnant patient and 17 patients without available key information in their medical records, we included 423 patients in the final analysis (Fig. 1). One hundred and ninety-one (43.3%) patients were middle-aged years (40–59 years), 119 (62.3%) of them had no obvious underlying diseases. Among these middle-aged patients without comorbidities, the degree of severity of COVID-19 was categorized as non-severe in 101 (84.9%) patients and severe in 18 (15.1%) patients. The clinical characteristics of middle-aged patients without comorbidities were shown in Table 1. The median age of these patients was 50 years (IQR, 40–54 years), and 77 (64.7%) patients were male. There was no significant difference in age, sex ratio and time from symptom onset to admission between the two groups. Fever (88.2%) and dry cough (67.7%) were the most common symptoms, followed by dyspnea (43.7%), expectoration (31.9%), fatigue (30.3%) and diarrhea (10.9%). High fever (> 39 ℃), expectoration, myalgia and dyspnea were more common in severe cases as compared with non-severe cases (p < 0.05). However, diarrhea was occurred only in non-severe cases. The comparison of clinical characteristics and outcome of COVID-19 patients in different age groups were shown in Additional file 1: Table S1.

**Table 1:** The characteristics of COVID-19 in middle-aged (40–59 years) patients without comorbidities on admission

Characteristics	All patients N = 119	Non-severe N = 101	Severe N = 18	*p* value^a^
Median age (IQR),yr	50 (45,54)	50 (45,55)	51(46,54)	0.970
Gender				
Female	42 (35.3)	37 (36.6)	5 (27.8)	NS
Male	77 (64.7)	64 (63.4)	13 (72.2)	0.469
Fever	38.5 (38.0,39.0)	38.5 (38.0,39.0)	39.0 (38.5,39.9)	0.002
37.3–38.0 ℃	27 (22.7)	25 (24.8)	2 (11.1)	0.203
38.1–39.0 ℃	56 (47.1)	47 (46.5)	9 (50.0)	0.786
> 39℃	22 (18.5)	15 (14.9)	7 (38.9)	0.016
Dry cough	76 (63.9)	68 (67.3)	8 (44.4)	0.063
Expectoration	38 (31.9)	28 (27.7)	10 (55.6)	0.020
Myalgia	7 (5.9)	4 (4.0)	3(16.7)	0.035
Fatigue	36 (30.3)	29(28.7)	7(38.9)	0.387
Dyspnea	52 (43.7)	38(37.6)	14(77.8)	0.002
Headache	5 (4.2)	5 (5.0)	0 (0)	0.229
Diarrhea	13 (10.9)	13 (12.9)	0 (0)	0.034
Heart rate, median (IQR),bpm	86 (80,96)	85 (80,96)	87 (84,96)	0.509
Respiratory rate, median (IQR)	20 (20,24)	20 (20,23)	21 (20,28)	0.095
Pulse oximetry %, median (IQR)	95 (86,98)	96 (93,98)	87 (80,93)	0.232
PaO_2_/FiO_2_ ratio, median (IQR)	362 (246,380)	383 (345,420)	313 (180,370)	0.423
Time from symptom onset to admission, median (IQR),d	7 (5,10)	6 (4,9)	9 (6,13)	0.797

Values are numbers (percentages) unless stated otherwise

COVID-19 coronavirus disease 2019**,** IQR interquartile range

^**a**^p values indicate differences between severe and non-severe. p < 0.05 was considered statistically significant

### Laboratory and radiologic findings of middle-aged (40–59 years) COVID-19 patients without comorbidities on admission

The laboratory and radiologic findings of middle-aged (40–59 years) COVID-19 patients without comorbidities on hospital admission were shown in Table 2. Compared with non-severe patients, the levels of white blood cell count, neutrophil count, ALT, LDH and procalcitonin were significantly higher in patients developing severe disease (all p < 0.05). Moreover, compared with non-severe patients, the neutrophil to lymphocyte ratio (14.9 vs. 4.34) and D-dimer (1.58 vs. 0.62) increased significantly in the severe patients (all p < 0.001). The lymphocyte count (0.53 vs. 1.17) and albumin (30.5 vs. 32.4) in severe patients were significantly lower than that in non-severe patients (all p < 0.05). The SOFA score in severe patients (median 2, IQR, 1–3) was significantly higher than that in non-severe patients (median 1, IQR, 0–2) on hospital admission (p = 0.002). A total of 110 (92.4%) patients had findings of bilateral infiltrates on radiographic imaging, while 9 (7.6%) patients had unilateral infiltrates on admission.

**Table 2:** The laboratory findings of COVID-19 in middle-aged (40–59 years) patients without comorbidities

Characteristics	All patients N = 119	Non-severe N = 101	Severe N = 18	*p* value^a^
White blood cell count (3.5–9.5 × 10^9^/L)	7.76 (6.15,10.0)	7.60 (6.11,9.03)	9.00 (6.73,11.3)	0.004
< 4 (n,%)	7 (5.9)	7 (6.9)	0 (0)	0.543
4–10 **(**n,%)	93 (78.2)	81 (80.2)	12 (66.7)	0.201
> 10 (n,%)	19 (16.0)	13 (12.9)	6 (33.3)	0.029
Neutrophil count (1.8–6.3 × 10^9^/L)	5.73 (4.66,7.11)	5.57 (4.47,6.91)	7.61 (6.08,9.34)	0.001
> 6.3 **(**n,%)	49 (41.2)	37 (36.6)	12 (66.7)	0.017
Lymphocyte count (1.1–3.2 × 10^9^/L)	1.05 (0.73,1.57)	1.17 (0.89,1.62)	0.53 (0.44,0.77)	< 0.001
< 0.8 (n,%)	36 (30.3)	22 (21.8)	14 (77.8)	< 0.001
NLR	4.97 (3.18,9.26)	4.34 (3.06,7.11)	14.9 (10.1,18.7)	< 0.001
Haemoglobin (316-354 g/L)	128 (119,136)	128 (119,136)	130 (122,134)	0.856
Platelet count (125–350 × 10^9^/L)	245 (191,302)	245 (192,312)	247 (167,273)	0.313
< 100 (n,%)	3 (2.5)	1 (1.0)	2 (11.1)	0.059
D-dimer (0–1.5 μg/ml)	0.70 (0.34,1.23)	0.62 (0.32,0.96)	1.58 (1.09,3.78)	< 0.001
> 1 **(**n,%)	36 (31.3)	22 (22.4)	14 (82.4)	< 0.001
ALT (9-50U/L)	31 (21,65)	29 (19,59)	55 (31,119)	0.021
Prealbumin (200–430 mg/L)	155 (104,219)	161 (106,225)	129 (99,165)	0.162
Albumin (40–55 g/L)	31.7 (29.8,35.8)	32.4 (30.0,36.1)	30.5 (27.8,31.7)	0.014
Bilirubin (0-26μmmol/L)	9.70 (6.70,14.3)	9.80 (6.85,13.7)	9.60 (6.48,17.2)	0.986
Troponin (0–28 pg/mL)	2.5 (0.9,4.3)	2.4 (0.8,4.2)	2.9 (1.3,5.5)	0.572
Total cholesterol (3.3–5.2mmoL/L)	3.83 (3.28,4.34)	3.81 (3.28,4.36)	3.95 (3.34,4.29)	0.978
Triglyceride (0.51–1.70mmoL/L)	1.42 (1.13,1.92)	1.35 (1.11,1.89)	1.48 (1.23,1.94)	0.487
Low density lipoprotein (2.1–3.37mmoL/L)	2.27 (1.83,2.78)	2.27 (1.83,2.78)	2.29 (1.81,2.72)	0.830
High density lipoprotein (1.04–1.55 mmoL/L)	0.92 (0.79,1.12)	0.95 (0.79,1.14)	0.89 (0.79,0.99)	0.477
Serum creatinine (44–97 μmol/L)	68.7 (55.9,77.8)	69.4 (56.4,78.7)	66.0 (53.7,69.6)	0.159
Creatine kinase (0–190U/L)	72.5 (43.0,123)	67.0 (44.0,124)	96.0 (39.5,121)	0.618
CK-MB (0–25U/L)	12.0 (10.0,16.0)	12.0 (9.0,15.0)	16.0 (10.8,18.0)	0.069
HS-CRP (0–5 mg/L)	27.7 (6.65,79.9)	21.2 (6.10,79.2)	45.2 (26.7,74.2)	0.108
LDH (120–250 U/L)	307 (225,363)	290 (214,352)	351 (299,454)	0.007
IL-6 (0–7 pg/ml)	8.33 (5.85,11.6)	8.38 (5.87,11.6)	7.62 (5.85,12.7)	0.669
Procalcitonin (0–0.05 ng/ml)	0.05 (0.05,0.05)	0.05 (0.05,0.05)	0.08 (0.05,0.10)	0.002
SOFA score, median (IQR)	1 (0,2)	1 (0,2)	2 (1,3)	0.002
Unilateral pneumonia^**b**^ (n, %)	9 (7.6)	9 (8.9)	0 (0)	0.352
Bilateral pneumonia^c^ (n,%)	110 (92.4)	92 (91.1)	18 (100)	0.188

Values are median (IQR) unless stated otherwise

COVID-19 coronavirus disease 2019, NLR neutrophil to lymphocyte ratio, ALT alanine amino transferase, CK-MB creatine kinase isoenzyme-MB, HS-CRP high sensitive c reaction protein, LDH lactate dehydrogenase, IL-6 interleukin-6, SOFA Sequential Organ Failure Assessment

^a^p values indicate differences between severe and non-severe. p < 0.05 was considered statistically significant

^b^Any patient with a chest radiograph or CT imaging of pulmonary infections manifested single lung shadowing

^c^Any patient with a chest radiograph or CT imaging of pulmonary infections manifested double lung shadowing

### The complications and outcomes of middle-aged (40–59 years) COVID-19 patients without comorbidities

Among the 119 patients who were discharged or died at the study end point, 11 (9.24%) were treated in the ICU, 18 (15.1%) received mechanical ventilation, 2 (1.68%) were treated with continuous renal replacement therapy, and 5 (4.2%) died (Table 3). ARDS (26, 21**.**8%) was the most common complications, followed by acute liver injury (16, 13**.**4%), septic shock (5, 4**.**3%), acute cardiac injury (4, 3**.**4%) and acute kidney injury (3, 2**.**5%). Severe patients yielded significantly higher rates of any complication as compared with non-severe patients. The median time from symptom onset to ARDS in severe and non-severe patients was 8 days (IQR, 7–12) and 10 days (IQR, 8–11), respectively. The median time from symptom onset to other complications was all about 2 weeks. Mortality rate in severe patients was 27.8%, while there was no death in the non-severe patients. The general characteristics and cause of death of 5 non-survived COVID-19 middle-aged patients without comorbidities were shown in Table 4. Severe ARDS was the main cause of death.

**Table 3:** The complications and outcomes of COVID-19 in middle-aged (40–59 years) patients without comorbidities

Characteristics	All patients N = 119	Non-severe N = 101	Severe N = 18	*p* Value^a^
ARDS	26 (21.8)	8 (7.9)	18 (100.0)	< 0.001
Sepsis shock	5 (4.2)	0 (0)	5 (27.8)	0.003
Acute liver injury	16 (13.4)	8 (7.9)	8 (44.4)	< 0.001
Acute kidney injury	3 (2.5)	1 (0.9)	2 (11.1)	0.059
Acute cardiac injury	4 (3.4)	0 (0)	4 (22.2)	< 0.001
Time from symptom onset to ARDS (IQR),d	9 (7,12)	10 (8,11)	8 (7,12)	0.368
Time from symptom onset to sepsis shock, median (IQR),d	12 (9,20)	–	12 (9,20)	NS
Time from symptom onset to acute liver injury, median (IQR),d	12 (9,14)	11 (8,14)	13 (11,14)	0.397
Time from symptom onset to acute kidney injury, median (IQR),d	11 (10,16)	8 (–)	16 (14,19)	0.221
Time from symptom onset to acute cardiac injury, median (IQR),d	15 (13,17)	–	15 (13,17)	NS
Time from symptom onset to discharge or death, median (IQR),d	22 (19,26)	25 (22,30)	21 (19,26)	0.171
ICU admission	11 (9.24)	–	11 (61.1)	NS
Mechanical ventilation	18 (15.1)	–	18 (100)	NS
Vasopressor therapy	5 (4.2)	–	5 (27.8)	NS
CRRT	2 (1.68)	–	2 (11.1)	NS
ECMO	0(0)	–	0 (0)	NS
Death	5 (4.2)	0 (0)	5 (27.8)	0.003

Values are numbers (percentages) unless stated otherwise

COVID-19 coronavirus disease 2019, ARDS acute respiratory distress syndrome**,** IQR inter quartile range, ICU intensive care unit, CRRT continuous renal replacement therapy, ECMO extracorporeal membrane oxygenation

^a^ p values indicate differences between severe and non-severe. p < 0.05 was considered statistically significant

**Table 4:** General characteristics and cause of death of 5 non-survived COVID-19 middle-aged patients without comorbidities

ID	Gender	Age	Time from symptom onset to admission, d	Time from symptom onset to death, d	NLR	D-dimer (μg/ml)	Cause of death
1	Male	51	6	14	25.68	3.78	Severe ARDS, sepsis shock, acute cardiac injury, acute kidney injury, cardiac arrest
2	Male	53	10	28	17.11	35.53	Severe ARDS, sepsis shock, gastrointestinal hemorrhage
3	Male	54	7	13	13.79	32.94	Severe ARDS, cardiac arrest
4	Male	55	11	16	19.12	2.06	Severe ARDS, acute cardiac injury, acute liver injury, cardiac arrest
5	Male	56	4	22	12.23	1.04	Severe ARDS, acute liver injury, cardiac arrest

COVID-19, coronavirus disease 2019; NLR, neutrophil to lymphocyte ratio; ARDS, acute respiratory distress syndrome

### Risk factors for severe COVID-19 of middle-aged patients without comorbidities

The results of univariate and multivariate logistic regression models assessing the relations between variables on admission and the development of severe COVID-19 were shown in Table 5. In univariable analysis, high fever, dyspnea, leucocytosis, lymphopenia, elevated NLR, lactate dehydrogenase, hypoalbuminemia, D-dimer greater than 1 μg/ml and higher SOFA score at admission were associated with the development of severe COVID-19. Additionally, multivariate logistic regression analysis revealed that the higher NLR (OR, 1.238, 95% CI 1.110–1.382, p < 0.001) and D-dimer greater than 1 μg/ml (OR, 16.079, 95%CI, 3.162–81.775, p = 0.001) on admission were the independent risk factors for the development of severe COVID-19 (Table 5).

**Table 5:** Logistic regression modeling evaluating risk factors for severe COVID-19 in middle-aged (40–59 years) patients without comorbidities

Variables	Univariate logistic regression			Multivariate logistic regression		
	OR	95% CI	*p* value^a^	OR	95% CI	*p* value^a^
Fever > 39℃	3.648	1.221–10.905	< 0.001			
Myalgia	4.850	0.986–23.846	0.052			
Dyspnea	5.803	1.780–18.919	0.004			
White blood cell count	1.229	1.049–1.440	0.011			
Neutrophil count	1.297	1.097–1.533	0.002			
Lymphocyte count	0.045	0.008–0.253	< 0.001			
NLR	1.245	1.132–1.368	< 0.001	1.238	1.110–1.382	< 0.001
Platelet count	0.996	0.990–1.002	0.156			
D-dimer > 1 μg/ml	16.121	4.246–61.210	< 0.001	16.079	3.162–81.775	0.001
ALT	1.006	0.999–1.013	0.088			
Albumin	0.828	0.710–0.965	0.016			
LDH	1.007	1.002–1.011	0.003			
Procalcitonin	1.753	0.106–28.863	0.695			
SOFA	1.854	1.268–2.711	0.001			

COVID-19 coronavirus disease 2019, OR Odds Ratio**,** CI confidence interval**,** NLR neutrophil to lymphocyte ratio, ALT alanine amino transferase, LDH lactate dehydrogenase, SOFA Sequential Organ Failure Assessment

^a^p < 0.05 was considered statistically significant

### Predictive performance of the NLR, D-dimer and combined index for the development of severe COVID-19

**Figure. 2:**
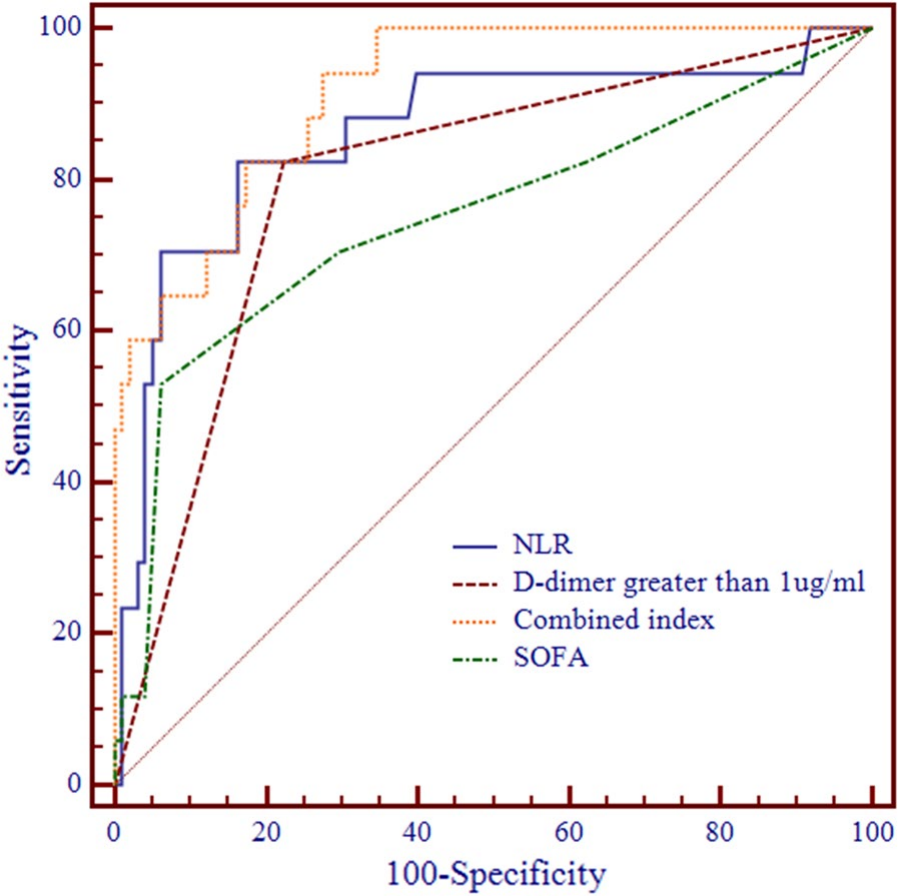
ROC curve analysis using the NLR, D-dimer, combined index and SOFA for predicting severe COVID-19 in middle-aged (40–59 years) patients without comorbidities. COVID-19 coronavirus disease 2019, AUC area under the curve, CI confidence interval, NLR neutrophil to lymphocyte ratio, SOFA Sequential Organ Failure Assessment, Combined index combined NLR and D-dimer > 1 μg/ml index

ROC curve analysis was used to analyze the predictive performance of the NLR, D-dimer and combined NLR and D-dimer (Fig. 2). As recent publications demonstrated that SOFA could well predict the severity and outcome of COVID-19, we compared the predictive performance of these risk factors and SOFA for the development of severe COVID-19 in middle-aged patients without underlying disease. The optimal cut-offs and corresponding sensitivity and specificity and AUC were listed in Table 6. The optimal cut-off value of NLR for predicting severe illness was 5.03, which yielded sensitivity and specificity of 88.2% and 66.2%, respectively. The optimal cut-off value of SOFA was 2, which resulted in sensitivity and specificity values of 70.6% and 70.4%, respectively. SOFA and NLR yielded an AUC of 0.750 (95% CI 0.602–0.987) and 0.862 (95% CI 0.751–0.973), respectively. However, there was no significant difference in the AUC between SOFA and NLR (Z = 1.325, p = 0.185). We further combined NLR and D-dimer higher than 1 μg/ml to draw another ROC curve, as shown in Fig. 2, yielding much greater discriminatory capacity for severe illness, with an AUC of 0.916 (95% CI 0.855–0.977). The Delong’s test showed that there was significant difference in the AUC between SOFA and combined index (z = 2.574, p = 0.010). These results demonstrated the prediction effect of the combined index was significantly better than that of SOFA.

**Table 6:** Predictive performance of NLR, D-dimer, combined index and SOFA for severe COVID-19 in middle-aged (40–59 years) patients without comorbidities

Variables	AUC	Cut-off	95%CI	Sensitivity	Specificity	*p* value^a^
NLR	0.862	5.03	0.751–0.973	88.2%	66.2%	< 0.001
D-dimer > 1 μg/ml	0.800	–	0.684–0.915	82.4%	77.6%	< 0.001
Combined index	0.916	–	0.855–0.977	82.4%	82.7%	< 0.001
SOFA	0.750	2.0	0.602–0.987	70.6%	70.4%	0.001

COVID-19 coronavirus disease 2019, AUC area under the curve, CI confidence interval, NLR neutrophil to lymphocyte ratio, Combined index combined NLR and D-dimer > 1 μg/ml, SOFA Sequential Organ Failure Assessment

## Discussion

To our knowledge, the present study is the first multicentre study to investigate the clinical characteristics, risk factors and predictors for severe illness in middle-aged COVID-19 patients without comorbidities. In this retrospective cohort study, the incidence of severe COVID-19 in middle-aged patients without comorbidities was significantly lower than that in elderly patients (15.1% vs. 57.1%) while higher than that in young patients (15.1% vs. 2.6%). In addition, the incidence of complications in this specific population was lower than that in general population except for ARDS and acute liver injury. We also found that elevated NLR and D-dimer levels on admission were risk factors for the development of severe COVID-19. In particular, the combination of NLR and D-dimer levels higher than 1 μg/ml had a good predictive value for severe COVID-19 in this specific population, even better than SOFA score.

In the middle-aged COVID-19 patients without comorbidities in this study, patients with high fever, dyspnea, elevated levels of NLR, LDH and D-dimer, as well as decreased ALB in early stage were more common in severe COVID-19. Compared to the overall COVID-19 patients (Additional file 1: Table S2), the proportion of severe cases, as well as the incidence of sepsis shock, acute cardiac injury, and other organ injury complications were lower in middle-aged COVID-19 patients. There was no difference in the incidence of acute liver injury between the two groups. Similar to previous studies, the prevalence of abnormal liver function tests (LFTs) was high in COVID-19 patients, whereas acute liver injury was usually mild, with limited clinical relevance and no treatment required [19–21].

Extensive studies have suggested that COVID-19 patients with any comorbidity were more likely to develop severe organ injury related to pre-existing diseases. A retrospective cohort study of 3,069 hospitalized patients with COVID-19 in US demonstrated that patients with cardiovascular disease **(**CVD) were more likely to have myocardial injury than patients without CVD (73.2% vs. 19.3%) [22]. Similarly, compared with COVID-19 patients without chronic obstructive pulmonary disease (COPD), patients with COPD were more likely to develop pulmonary bacterial or fungal coinfection (20.0% vs*.*5.9%), ARDS (20.0% vs. 7.3%), and septic shock (14.0% vs. 2.3%) [23]. In addition, patients with pre-existing renal diseases were more susceptible to develop renal complications induced by COVID-19. A meta-analysis of 22 studies also showed that groups with higher prevalence of pre-existing CKD have higher incidence of AKI [24], and the pre-existing CKD was associated with severe illness or death in COVID-19 [25]. Thus, the presence of these comorbidities might have increased the risk of mortality independent of COVID-19 infection. In other words, some patients may die from the deterioration of comorbidities that induced by SARS-CoV-2 infection, rather than the direct damage of organs caused by SARS-CoV-2.

Among 5 deceased patients, in addition to severe ARDS, elevated D-dimer and cardiac arrest were the most common causes of death. Unfortunately, the lack of autopsy made it impossible to determine the actual causes of death. However, the possibility of cardiac arrest induced by severe hypoxemia and fatal embolism events (e.g. pulmonary embolism) should be highly suspected. Accumulating evidence supported that the hypercoagulability of SARS-CoV-2 involved a unique mechanisms of thrombo-inflammation triggered by viral infection, originating in the pulmonary vasculature [26]. Moreover, the incidence of pulmonary embolism was high in severe patients with COVID-19. In a larger study in the Netherlands, the cumulative incidence of thrombotic events in184 ICU patients with COVID-19 was 49%, with pulmonary embolism being the most frequent (65/75, 87%) [28]. Middeldorp and colleagues also reported a high incidence of thrombotic complications in their ICU patient population (7-day and 14-day cumulative incidence of 26% and 47%, respectively), although all patients initially received standard care of thromboprophylaxis [29]. Our results suggested that lung and coagulation system suffered the most serious attack by SARS-CoV-2 in middle-aged COVID-19 patients without comorbidities, while other organs were less damaged. Therefore, for this specific patient population, more attention should be paid to the monitoring and early treatment of respiratory and coagulation abnormalities.

In the present study, the higher NLR and D-dimer levels greater than 1 μg/ml were independent risk factors for the development of severe COVID-19. The NLR reflects the status of systematic inflammation and immune response**.** Neutropenia represents the aggravation of physiological stress and inflammatory response, and lymphopenia reflects the suppression of immune function [26, 30]. Dysregulated inflammatory and immune responses played an important role in the aggravation of COVID-19 [30–32]. Consistent with our results, in a meta-analysis of 5 studies from China with 828 patients, NLR was found to increase significantly in patients with severe diseases (standardized mean difference = 2.404, 95% CI 0.98–3.82) [33]. Besides COVID-19, the increased NLR has been shown to have strong association with the severity of many other diseases, including septic shock [34], tumor [35], and bacterial infection [36]. Moreover, numerous studies have shown that the hypercoagulable state induced by COVID-19 was associated with poor outcomes of patients [2, 37, 38]. Consistent with these recent studies, we found in this study that D-dimer higher than 1 μg/ml on admission was as much as 16.079 times (95% CI 3.162–81.775) more likely to develop severe COVID-19 than those with D-dimer lower than 1 μg/ml. In a recent meta-analysis, Wu, et al. demonstrated that higher C reaction protein (CRP) levels were commonly observed in COVID-19 patients who developed thromboembolic events, and the thromboembolic events were also associated with adverse outcomes [27]. The association between acute inflammation and thromboembolic events has been indicated by numerous studies [26, 28, 29]. In addition, endothelial activation or dysfunction, and complement activation might be all involved in the hypercoagulable state in COVID-19 [39, 40].

We also found in our study that the combined NLR and D-dimer index was a good prognostic biomarker for the development of severe COVID-19, even better than SOFA score. Our results showed that NLR alone yielded a relatively high AUC (0.862, 95% CI 0.751–0.973) to predict the development of severe COVID-19, while the specificity was just 66.2%. The combined use of D-dimer and NLR not only yielded a significantly elevated AUC of 0**.**916 (95% CI 0.855–0.977; p < 0.001), but also resulted in a greatly increased specificity from 66.2% to 82.7%. The SOFA score was a morbidity severity score and was originally designed to focus on organ dysfunction and morbidity [41]. Increasing evidences have suggested that SOFA score could well predict the severity and outcome of the disease [42, 43], including sepsis, septic shock [12], as well as COVID-19 [2]. In this study, although SOFA yielded a AUC (0.750, 95% CI 0.602–0.987) with 70.6% sensitivity and 70.4% specificity, the statistical results indicated that the combined index was significantly better than SOFA for predicting the incidence of severe illness in COVID-19 **(**z = 2.574, p = 0.010). In addition, compared with the overall patient population, the prediction for severe COVID-19 by this combined index showed higher sensitivity (82.4%) and specificity (82.7%) in middle-aged patients without comorbidities. In a study of 96 patients, Yang, et al. demonstrated that the optimal cut-off value of NLR for predicting severe COVID-19 was 3.3, which yielded sensitivity and specificity of 63.6% and 88.0%, respectively [44]. Similarly, in another analysis of 301 patients, a NLR at 2.973 was associated with the progression of COVID-19, which only yielded an AUC of 0.734, with sensitivity and specificity of 75.8% and 66.8%, respectively [45]. Most importantly, compared with SOFA score consisting of 6 variables, NLR and D-dimer could be obtained much easier and quicker by routine hematology. Therefore, combined NLR and D-dimer index might be an easy-to-use and reliable predictor for the severity of the middle-aged COVID-19 patients without comorbidities. Remarkably, many other prognostic factors are widely investigated in patients with COVID-19, such as CRP and other inflammatory biomarkers which correlates to disease severity. Recently a systematic review and a meta-analysis enhanced these data in COVID-19 patients. Izcovich, et al. included 207 studies and found high or moderate certainly that 49 variables, including high interleukin-6 (IL-6), high blood lactate dehydrogenase (LDH) and many other indicators, could provide valuable prognostic information on mortality and/or severe disease in COVID-19 [46]. In addition, in a meta-analysis of 5350 COVID-19 patients from 25 studies, Huang, et al. concluded that an elevated serum CRP, procalcitonin, D-dimer, and ferritin were associated with a poor outcome in COVID-19 [47]. Therefore, in the face of more and more COVID-19 related risk factors, how to select the most effective and convenient predictors in specific populations still need more research.

Our study has some limitations. First, due to the retrospective study design, not all laboratory tests were done for all patients, especially the detection of immune related indicators. Although lymphocyte count could partly reflect the suppression of immune function, there was still a lack of comprehensive understanding of the patient's immune status. Second, the past history was provided by the patients or their relatives. Some patients might have unknown comorbidities due to the lack of previous basic medical data. However, these patients had a high self-awareness rate of hypertension, diabetes and other common diseases, owing to most of them came from cities and towns and received regular screening for common diseases. Third, as a multicentre study, the data in the study were from three different hospitals, which might lead to differences in testing results. However, the data in this study were all clinical routine test items. With the continuous promotion of external quality evaluation and standardization of clinical laboratory, the routine test items in most hospitals in China had reached a high degree of standardization.

## Conclusions

In summary, our study revealed that the lung and coagulation system suffered the most serious attack by SARS-CoV-2 in middle-aged COVID-19 patients without comorbidities while other organs were less damaged. More attention should be paid to the monitoring and early treatment of respiratory and coagulation abnormalities in this specific population. In addition, the combined NLR and D-dimer higher than 1 μg/ml index might be a potential and reliable predictor for the incidence of severe illness in this specific patient with COVID-19, which could guide clinicians on early classification and management of patients, thereby relieving the shortage of medical resource. However, it is warranted to validate the reliability of the predictor in larger sample COVID-19 patients.

## Supplementary Information

**Additional file 1: Table S1.** The comparison characteristics and outcomes of COVID-19 patients without comorbidities in different age groups. Values are median (IQR) unless stated otherwise. **Table S2.** The complications and outcomes of COVID-19 in all adult patients and middle-aged (40-59 years) patients without comorbidities. Values are numbers (percentages) unless stated otherwise.

## Data Availability

All data analyzed during the current study are included in this article.
