# Supplementary material for: Risk factors for severe COVID-19 in middle-aged patients without comorbidities: a multicentre retrospective study
Source: J Transl Med. 2020 Dec 7;18:461. doi: 10.1186/s12967-020-02655-8 (PMC7719726; doi:10.1186/s12967-020-02655-8)
Supplement: Supplementary file 1 — Additional file 1: Table S1. The comparison characteristics and outcomes of COVID-19 patients without comorbidities in different age groups. Values are median (IQR) unless stated otherwise. Table S2. The complications and outcomes of COVID-19 in all adult patients and middle-aged (40-59 years) patients without comorbidities. Values are numbers (percentages) unless stated otherwise. [file 12967_2020_2655_MOESM1_ESM.docx]

**Table S1 The comparison** characteristics **and outcomes of COVID-19 patients without comorbidities in different age groups.** Values are median (IQR) unless stated otherwise.

| **Variables** | **All patients**  **N=271** | **19-39y**  **N=117** | **40-59y**  **N=119** | **Over 60y**  **N=35** | ***p* Value^a^** |
| --- | --- | --- | --- | --- | --- |
| Median age (IQR),yr | 42(35,52) | 32(29,36) | 50(45,54) | 64(63,67) | <0.001 |
| Male, n(%) | 156(57.6) | 60(51.3) | 78(65.5) | 18(51.4) | 0.208 |
| Time from symptom onset to admission, median (IQR),d | 10(6,14) | 9(7,12) | 7(5,10) | 12(7,15) | 0.045 |
| Laboratory findings on admission |  |  |  |  |  |
| White blood cell count  (3.5-9.5×10^9^/L) | 6.90  (5.70,8.48) | 6.33  (4.74,7.90) | 7.76  (6.15,10.0) | 5.68  (4.69,7.50) | <0.001 |
| Neutrophil count  (1.8-6.3×10^9^/L) | 5.15  (3.56,6.91) | 4.25  (2.77,5.76) | 5.73  (4.66,7.11) | 4.51  (3.39,5.35) | <0.001 |
| Lymphocyte count  (1.1-3.2×10^9^/L) | 1.24  (0.90,1.73) | 1.45  (1.15,1.85) | 1.24  (0.87,1.61) | 0.95  (0.77,1.28) | <0.001 |
| NLR | 4.06  (2.32,7.55) | 2.25  (1.74,4.87) | 4.97  (3.18,9.26) | 3.82  (2.48,6.32) | <0.001 |
| Platelet count  (125-350×10^9^/L) | 243  (192,300) | 254  (219,298) | 245  (191,302) | 197  (168,299) | 0.053 |
| D-dimer  (0-1.5μg/ml) | 0.66  (0.36,1.17) | 0.56  (0.34,1.07) | 0.70  (0.34,1.23) | 0.72  (0.48,2.06) | 0.216 |
| HS-CRP  (0-5mg/L) | 24.8  (4.0,71.9) | 5.20  (0.95,29.5) | 27.7  (6.65,79.9) | 44.1  (14.6,97) | 0.002 |
| IL-6  (0-7pg/ml) | 8.38  (6.13,11.1) | 7.93  (6.38,10.1) | 8.33  (5.85,11.6) | 8.99  (7.52,13.2) | 0.456 |
| LDH  (120-250 U/L) | 287  (226,359) | 273  (226,342) | 306  (225,363) | 284  (242,389) | 0.640 |
| ALT  (9-50U/L) | 30.0  (19.3,58.5) | 32.0  (18.5,60.3) | 31.0  (20.8,65.0) | 25.0  (20.3,33.0) | 0.144 |
| Bilirubin (0-26μmmol/L) | 10.7  (7.8,14.8) | 11.0  (8.03,15.7) | 9.70  (6.70,14.3) | 11.8  (9.0,15.0) | 0.123 |
| Prealbumin  (200-430mg/L) | 159  (106,211) | 171  (132,206) | 155  (104,219) | 94.0  (73.5,172) | 0.010 |
| Albumin (40-55g/L) | 31.8  (29.0,35.6) | 31.7  (27.7,35.8) | 31.7  (29.8,35.8) | 32.3  (29.2,34.5) | 0.702 |
| Serum creatinine  (44-97μmol/L) | 69.0  (55.2,78.7) | 70.0  (55.2,78.8) | 68.7  (55.9,77.8) | 66.3  (54.7,84.2) | 0.935 |
| CK-MB (0-25U/L) | 12.0  (9.0,17.0) | 13.0  (9.0,18.5) | 12.0  (10.0,16.0) | 11.0  (9.0,18.0) | 0.388 |
| Troponin (0-28pg/mL) | 2.5(0.7,4.9) | 1.8(0.2,3.2) | 2.5(0.9,4.3) | 4.9(2.9,6.7) | 0.002 |
| Total cholesterol  (3.3-5.2mmoL/L) | 3.67  (3.25,4.25) | 3.57  (3.12,4.05) | 3.82  (3.28,4.34) | 3.72  (3.32,4.26) | 0.101 |
| Triglyceride  (0.51-1.70mmoL/L) | 0.94  (0.79,1.13) | 0.85  (0.76,1.05) | 0.92  (0.79,1.12) | 1.06  (0.97,1.14) | 0.003 |
| Low density lipoprotein  (2.1-3.37mmoL/L) | 2.23  (1.80,2.70) | 2.24  (1.62,2.50) | 2.27  (1.83,2.78) | 2.0  (1.81,2.64) | 0.659 |
| High density lipoprotein  (1.04-1.55mmoL/L) | 1.50  (1.11,1.96) | 1.71  (1.41,2.11) | 1.42  (1.13,1.92) | 1.21  (0.85,1.74) | 0.009 |
| SOFA | 1(0,1) | 0(0,1) | 1(0,2) | 2(0.5,2.5) | <0.001 |
| Complications and outcomes |  |  |  |  |  |
| ARDS | 49(18.1) | 3(2.6) | 26(21.8) | 20(57.1) | <0.001 |
| Time from symptom onset to ARDS (IQR),d | 8(6,12) | 7(6,11) | 9(7,12) | 7(5,10) | 0.461 |
| Sepsis shock | 7(2.6) | 0(0) | 5(4.2) | 2(5.7) | 0.056 |
| Time from symptom onset to sepsis shock, median (IQR),d | 14(12,20) | - | 12(9,20) | 18(15,25) | 0.001 |
| Acute liver injury | 32(11.8) | 12(10.3) | 16(13.4) | 4(11.4) | 0.393 |
| Time from symptom onset to acute liver injury, median (IQR),d | 13(10,16) | 16(10,23) | 12(9,14) | 11(10,16) | 0.783 |
| Acute kidney injury | 5(1.8) | 0(0) | 3(2.5) | 2(5.7) | 0.097 |
| Time from symptom onset to acute kidney injury, median (IQR),d | 12(10,18) | - | 11(10,16) | 14(12,25) | 0.439 |
| Acute cardiac injury | 7(2.6) | 0(0) | 4(3.4) | 3(8.6) | 0.023 |
| Time from symptom onset to acute cardiac injury, median (IQR),d | 15(13,20) | - | 15(13,17) | 16(10,24) | 0.658 |
| Severe | 41(15.1) | 3(2.6) | 18(15.9) | 20(57.1) | <0.001 |
| Death | 11(4.1) | 0(0) | 5(3.97) | 6(17.1) | <0.001 |

COVID-19 coronavirus disease 2019, NLR neutrophil to lymphocyte ratio, ALT alanine amino transferase, CK-MB creatine kinase isoenzyme-MB, HS-CRP high sensitive c reaction protein, LDH lactate dehydrogenase, IL-6 interleukin-6.

^a^p values indicate differences among three age groups (19-39y, 40-59y and over 60y). p < 0.05 was considered statistically significant.

Table S2 The **complications and outcomes of** **COVID-19 in all adult patients and middle-aged (40-59 years) patients without comorbidities.** Values are numbers (percentages) unless stated otherwise.

| Characteristics | **Middle-aged patients without comorbidities**  **N=119** | **All adult patients**  **N=413** | ***p* Value^a^** |
| --- | --- | --- | --- |
| ARDS | 26(21.8) | 102(24.7) | 0.543 |
| Sepsis shock | 5(4.2) | 34(8.2) | 0.019 |
| Acute liver injury | 16(13.4) | 51(12.3) | 0.709 |
| Acute kidney injury | 3(2.5) | 28(6.8) | 0.104 |
| Acute cardiac injury | 4(3.4) | 43(10.4) | 0.012 |
| Time from symptom onset to ARDS (IQR),d | 9(7,12) | 12(8,16) | 0.103 |
| Time from symptom onset to sepsis shock, median (IQR),d | 12(9,20) | 18(14,23) | 0.529 |
| Time from symptom onset to acute liver injury, median (IQR),d | 12(9,14) | 16(11,21) | 0.073 |
| Time from symptom onset to acute kidney injury, median (IQR),d | 11(10,16) | 19(13,24) | 0.239 |
| Time from symptom onset to acute cardiac injury, median (IQR),d | 15(13,17) | 16(10,22) | 0.718 |
| Time from symptom onset to discharge or death, median (IQR),d | 22(19,26) | 25(22,30) | 0.732 |
| Severe | 18(15.1) | 91(22.0) | 0.100 |
| Death | 5(4.2) | 31(7.5) | 0.003 |

COVID-19 coronavirus disease 2019, ARDS acute respiratory distress syndrome, IQR inter quartile range.

^a^p values indicate differences between middle-aged patients without comorbidities and all adult patients. p < 0.05 was considered statistically significant.
